# Ambulatory Human Gait Phase Detection Using Wearable Inertial Sensors and Hidden Markov Model

**DOI:** 10.3390/s21041347

**Published:** 2021-02-14

**Authors:** Long Liu, Huihui Wang, Haorui Li, Jiayi Liu, Sen Qiu, Hongyu Zhao, Xiangyang Guo

**Affiliations:** 1 Department of Electrical & Information Engineering, Dalian Neusoft University of Information, Dalian 116023, China; liulong@neusoft.edu.cn; 2 School of Control Science and Engineering, Dalian University of Technology, Dalian 116024, China; 17303779346@mail.dlut.edu.cn (H.L.); Liujiayi@mail.dlut.edu.cn (J.L.); zhaohy@dlut.edu.cn (H.Z.); 15736873491@163.com (X.G.); 3 School of Fundamental Education, Dalian Neusoft University of Information, Dalian 116023, China; wanghuihui@neusoft.edu.cn

**Keywords:** body sensor network, gait analysis, gyroscope, information fusion, hidden Markov model

## Abstract

Gait analysis, as a common inspection method for human gait, can provide a series of kinematics, dynamics and other parameters through instrumental measurement. In recent years, gait analysis has been gradually applied to the diagnosis of diseases, the evaluation of orthopedic surgery and rehabilitation progress, especially, gait phase abnormality can be used as a clinical diagnostic indicator of Alzheimer Disease and Parkinson Disease, which usually show varying degrees of gait phase abnormality. This research proposed an inertial sensor based gait analysis method. Smoothed and filtered angular velocity signal was chosen as the input data of the 15-dimensional temporal characteristic feature. Hidden Markov Model and parameter adaptive model are used to segment gait phases. Experimental results show that the proposed model based on HMM and parameter adaptation achieves good recognition rate in gait phases segmentation compared to other classification models, and the recognition results of gait phase are consistent with ground truth. The proposed wearable device used for data collection can be embedded on the shoe, which can not only collect patients’ gait data stably and reliably, ensuring the integrity and objectivity of gait data, but also collect data in daily scene and ambulatory outdoor environment.

## 1. Introduction

Walking is one of the most common physical activities for humans and plays an important role in our daily activities. It can be performed in a variety of ways and directions, and is also an energy efficient method of mobility. For most people, walking is completely subconscious. In patients with neurological conditions such as stroke, this can be altered by gait abnormalities, which are usually caused by motor or sensory disorder. It is necessary to conduct specific rehabilitation exercise to deal with gait abnormalities, and the detection and tracking of gait can be of great help to patient recovery. Gait characterization and phase classification are widely used in the field of medical diagnosis to assess and detect the balance ability, which can be used for gait-based identification, robot control for artificial limbs and humanoid robots [1,2,3,4,5,6,7,8]. Researches about Micro-Electro-Mechanical Systems (MEMS) have developed rapidly over the past decade, enabling the development of computer communication devices, high-performance physical sensors, and especially inertial sensors. These sensors are characterized by their large memory capacity, small size and low cost, and it is due to these characteristics that they are widely used in various areas [9,10,11,12,13,14,15,16,17,18,19,20,21,22].

## 2. Related Works

According to the situation of foot contact with the ground, gait phase can be divided into two stages: support phase(ST) and swing phase(SW). SW and ST phases can also be divided into several subphases. The result is a model with three to eight phases. The four-stage division subdivides the support phase into four stages: Heel-strike (HS), Foot-flat (FF), Heel-off (HO) and Toe-off (TO). The time occupied by each stage is as follows: (1) HS to full FF, accounts for approximately 10% of the gait cycle. (2) Plantar Fascia FF to heel-to-ground (HF), accounts for approximately 35% of the gait cycle. (3) HF to toe-off (TF), accounts for approximately 15% of the gait cycle. (4) TO to HS in the next cycle, which accounts for about 40% of the gait cycle.

The hidden Markov model is a model of statistical analysis [23,24,25], which is used to describe Markov processes containing hidden parameters. Hidden Markov, as a typical statistical analysis model, is outstanding in solving problems in time series. Therefore, it has been widely studied in many areas of time series problem solving, and hidden Markov models are very useful in the analysis and processing of modeling problems. It has great advantages in modeling, such as modeling in the spatial direction. It also has advantages in the analysis of modeling directions, such as in solving the problem of modeling and analysis of non-stationary waveforms. The normal gait behavior of the human body is cyclical not only in time but also in space. It is because of the hidden Markov’s outstanding advantage in resolving cyclic information and the cyclic and regular nature of the human gait that both the relationship is a high match between the method and the problem, so in this paper we decided to adopt this model to solve the problem of gait stage division. The stages of gait behavior can be treated as the state of a Markov chain, and the data obtained by the acquisition device is extracted to obtain the eigenvalues by feature extraction, putting these eigenvalues correspond to the output observations of the Markov model.

Since human gait has a certain regularity and periodicity, many researchers have started to use hidden Markov models for gait behavior recognition (e.g., gait used to distinguish actions under different behaviors, gait used to identify different people for identification, and determining pathological gait) [26,27,28,29,30,31].

After the success of gait behavior recognition, some researchers began to study the hidden Markov model for gait stage delineation. On the issue of gait stage classification, the different problems solved by the researchers and the different types of gait data collected (including the collection of different data locations and different acquisition devices) make many differences in the problem of gait stage classification.

**Figure 1:**
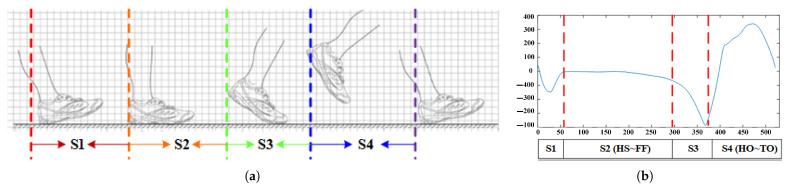
Traditional gait analysis methods (**a**) Correspondence between gait phases and Markov chain state (**b**) Correspondence between angular velocity and gait phases.

In this paper, it is hoped that a single inertial sensor can be used to obtain gait phase recognition with higher accuracy. Figure 1a presents the correlation between the gait stage and the Markov chain and Figure 1b divides a complete period of gait into four stages based on the angular velocity signal from compact inertial sensor.

The division of gait phases in this paper is based on the angular velocity collected at the toe and divides a complete gait cycle into four stages. The transitions between these four stages are carried out from left to right, and there is no jump transition, the gait stage in this paper with a Markov chain representation containing four states. The states corresponding to the four stages of the locomotor gait cannot be directly observed and are directly obtained as the angular velocity measured by the sensor, so we map of states to outputs can be achieved with the help of Gaussian probability or Gaussian mixed probability models to achieve gait stage division.

Wearable device-based gait phase segmentation and adaptive recognition have become a useful tool for quantitative medical diagnosis and patient recovery evaluation [15,32,33,34]. Human gait behavior shows regularity and periodicity, at the same time, since different individuals, environments, and individual states result in gait diversity, which brings a great deal of uncertainty. Researchers have been committed to accurately describing gait and improving the adaptability of the proposed gait analysis model [33,35,36,37,38,39,40,41]. In this paper, we mainly focus on the identification of the different phases of a single gait cycle, and the various phases of human gait reflect the individual health issues, which makes it useful in the field of diagnosis and guidance for medical rehabilitation.

The rest of this paper is summarized as follows: Section 3 analyzes the hidden Markov model. Section 4 describes the proposed wearable sensor system and the gait phase segmentation methodology. Experimental results are shown in Section 5. Section 6 discusses the proposed hidden Markov model and summarizes this paper.

## 3. Analysis of Hidden Markov Model

### 3.1. Analysis of HMM Theory

HMM usually consists of five parts: hidden state, model output value, initial state probability, transition probability between States and output probability distribution. The hidden state is usually represented by *S*, which is the actual requirement of the model, and usually can not be obtained by direct observation; the observable output is represented by *O*, which is the observed output of the model, and associated with the hidden state, can be regarded as the external performance of the hidden state; the probability matrix π of the initial state represents the probability of each state at the initial time; the transition between the hidden states The probability *A* is the transition probability between the hidden states, and the output probability matrix *B* is the probability that the corresponding output of one of the hidden states is an observed output. HMM is usually represented by $\theta =\left(\pi ,A,B\right)$.

The specific hidden Markov model can be described by five parts of the model:

(1) *N*: The number of states contained in a model is usually determined before the model is built. Suppose that the state at time *t* is $q_{t}$, then $q_{t}\in \left\{s_{1},s_{2},\cdots ,s_{N}\right\}$.
(2) *M*: The number of observation values corresponding to each state in the model (when the output observation value is discrete value), if the observation value of the model at time *t* is $o_{t}$, then $o_{t}\in \left\{v_{1},v_{2},\cdots ,v_{M}\right\}$.
(3) *A*: The state transition probability matrix of the model is $A={\left(a_{ij}\right)}_{N\times N}$. If the state of the model at time *t* is $q_{t}=s_{i}$, the transition probability can be expressed as $P\left(q_{t+1}=s_{j}|q_{t}=s_{i}\right)=a_{ij}$, and the state at time t+1 is $q_{t+1}=s_{j}$, where 1⩽t⩽T, *T* is the length of the model output observation value and 1⩽i,j⩽N. Meanwhile, the state transition matrix *A* satisfies $\sum_{j=1}^{N}a_{ij}=1,1⩽i,j⩽N$.
(4) $B={\left(b_{j}\left(k\right)\right)}_{N\times M}$: If the state of the model at a certain time is $q_{t}=s_{j}$ and the output observation value is $o_{t}=v_{k}$, the relationship between the state and the observation value can be expressed as $P\left(o_{t}=v_{k}|q_{t}=s_{j}\right)=b_{j}\left(o_{k}\right),1⩽j⩽N,1⩽k⩽M$, and the observation value probability matrix needs to meet $b_{j}\left(k\right)=1,1⩽k⩽M$.
(5) π: The probability of occurrence of each state in HMM model at the first time, $\pi =\left(\pi_{1},\pi_{2},\cdots ,\pi_{N}\right)$, when the initial state is $s_{i}$, can be expressed as $p\left(q_{1}=s_{i}\right)=\pi_{i},1⩽i⩽N$. The initial state probability needs to satisfy $\sum_{i=1}^{N}\pi_{i}=1$.

HMM contains two stochastic processes. The first process is Markov chain, which contains the initial state probability π and the state transition probability matrix *A* of HMM, and describes the state persistence and transition process, which is implicit. The second stochastic process describes the corresponding statistical relationship between the output state of Markov chain and the output of HMM model, which is described by the output probability matrix *B*.

There are two presuppositions to use HMM: homogeneous Markov hypothesis and independent observation hypothesis. In the homogeneous Markov hypothesis, the state of any time is only related to the state of the previous time of the current time, and has nothing to do with the state and output of other times. In the observation independence hypothesis, the corresponding output value at any time is only related to the state at the current time, and has nothing to do with the state and observation value at other times. The three problems of HMM and the corresponding algorithms are carried out under the premise of these two assumptions.

### 3.2. Implementation of EM Algorithm

**Figure 2:**
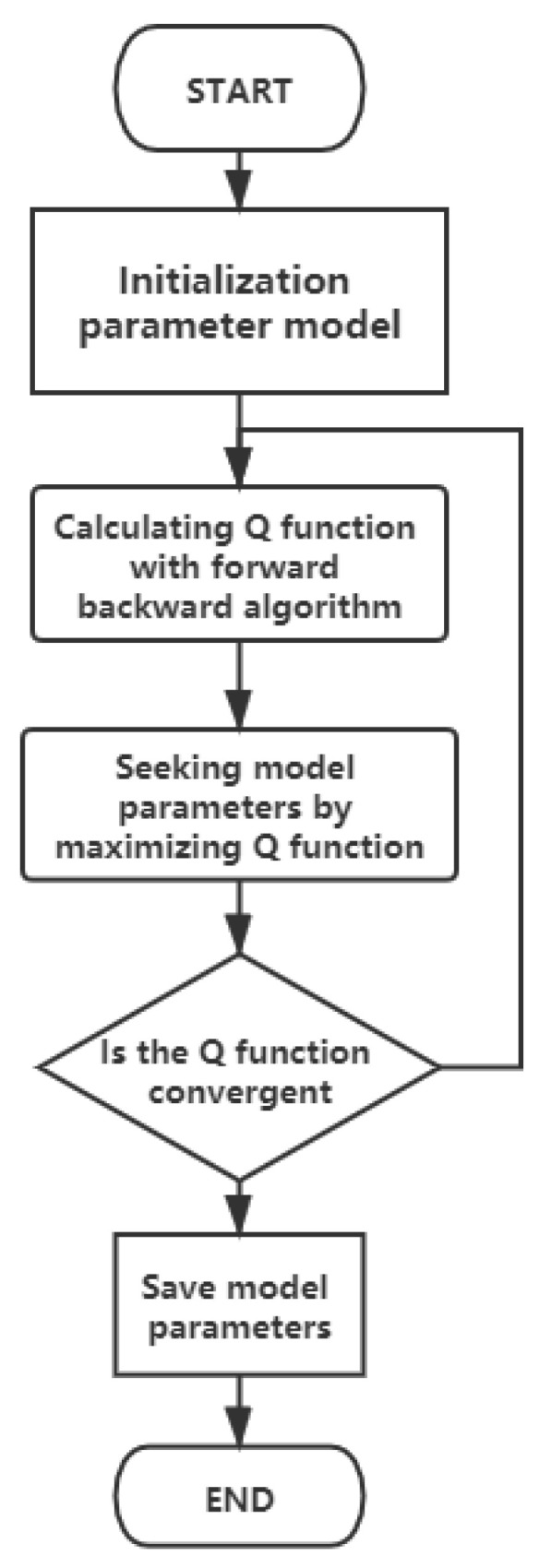
EM algorithm flow chart.

In the parameter solving problem of HMM, the model parameters are unknown and the output sequence is o. the model parameters that can make the probability of output sequence o maximum are obtained. If the variables in the probability model can be directly observed, the maximum likelihood estimation (MLE) method or Bayesian estimation method is usually used to calculate from the given data. Because HMM model contains hidden variables, we can not use these two methods directly. EM algorithm keeps approaching the optimal solution through iteration. It uses maximum likelihood estimation to solve the parameters of the model with hidden variables. Each iteration of EM algorithm consists of two steps. The first step is to find the expected joint probability expectation e, and the second step is to find the model parameters when the expectation reaches the maximum value, which is called the expected maximum algorithm. Figure 2 present the flow chart of the proposed EM algorithm.

The first step is usually to find the *Q* function, and the form of *Q* function is different according to the distribution of output sequence. The general formula of *Q* is:

(1) $$Q\left(\theta ,\theta^{old}\right)=\sum_{I}p\left(I,O|\theta^{old}\right)\ln p\left(I,O|\theta \right)$$

When the output observations are discrete distribution $B=\left\{v_{i}\right\}$, 1⩽i⩽M, where *M* is the number of discrete observations; when the output observations obey Gaussian distribution $B=\left\{\mu_{i},\sum i\right\},i=1,\cdots ,N$; when the output observations obey Gaussian mixture distribution, $B=\left\{B_{ij,}\mu_{ij},\sum ij\right\},1⩽i⩽N;1⩽j⩽M$, where *M* is used to represent the number of Gaussian distributions corresponding to the Gaussian mixture model corresponding to each state.

If the output distribution of HMM is discrete, the *Q* function of the model can be decomposed into three parts, each part is only related to a single model parameter.

(2) $$\begin{array}{cc} Q\left(\theta ,\theta^{old}\right)\; & =\sum_{i=1}^{N}p\left(q_{1}=s_{i},O|\theta^{old}\right)\ln\pi_{k}\\ \; & +\sum_{t=1}^{T-1}\sum_{i=1}^{N}\sum_{j=1}^{N}p\left(q_{t}=s_{i},q_{t+1}=s_{j},O|\theta^{old}\right)\ln a_{jk}\\ \; & +\sum_{t=1}^{T}\sum_{i=1}^{N}p\left(q_{t}=s_{i},O|\theta^{old}\right)\ln p\left(o_{t}|q_{t}=s_{i,\theta }\right) \end{array}$$

In order to simplify the calculation process, variables are introduced:

(3) $$\gamma_{t}\left(i\right)=\frac{\alpha_{t}\left(i\right)\beta_{t}\left(i\right)}{P\left(O|\theta \right)}=\frac{\alpha_{t}\left(i\right)\beta_{t}\left(i\right)}{\sum_{i=1}^{N}\sum_{j=1}^{N}\alpha_{t}\left(j\right)\beta_{t}\left(j\right)}$$

Let

(4) $$\xi_{t}\left(i,j\right)=p\left(q_{t}=s_{i},q_{t+1}=s_{j}|O,\theta \right)\;\;\left(1⩽t⩽T,1⩽i,j⩽N\right)$$

The expression can also be deduced by forward backward algorithm:

(5) $$\xi_{t}\left(i,j\right)=\frac{\alpha_{t}\left(i\right)a_{ij}b_{j}\left(o_{t+1}\right)\beta_{t+1}\left(j\right)}{\sum_{i=1}^{N}\sum_{j=1}^{N}\alpha_{t}\left(i\right)a_{ij}b_{j}\left(o_{t+1}\right)\beta_{t+1}\left(j\right)}\;\;$$

The second step is to find the hidden Markov model parameter θ when the *Q* function is maximized, and to solve the three model parameters by maximum likelihood method for the three terms of Equation (2). The parameter π of probability model are known by solving the first term.

(6) $$\sum_{j=1}^{K}\pi_{i}=1$$

Then we can get the Lagrange function of the first term as follows:

(7) $$L_{1}\left(\pi ,\theta^{old},\lambda \right)=\sum_{i=1}^{N}p\left(q_{1}=s_{i},O\right)\ln\pi_{i}+\lambda \left(\sum_{j=1}^{K}\pi_{i}-1\right)$$

The partial derivative of $\pi_{i}$ is calculated and set to 0, we can be concluded that:

(8) $$\pi_{i}=\gamma_{1}\left(i\right),i=1,\cdots ,N$$

According to the second term, the parameter state transition matrix *A* of probability model is solved, the known state transition matrix satisfies:

(9) $$\sum_{j=1}^{N}a_{ij}=1,j=1,\cdots ,N$$

Then the Lagrange function of the second term is:

(10) $$L_{2}\left(A,\theta^{old},\lambda_{1},\cdots \lambda_{N}\right)=\sum_{t=1}^{T-1}\sum_{i=1}^{N}\sum_{j=1}^{N}p\left(q_{t}=s_{i},q_{t+1}=s_{j},O|\theta^{old}\right)\ln\left(a_{jk}\right)+\sum_{j=1}^{N}\lambda_{j}\left(\sum_{k=1}^{M}b_{j}\left(k\right)-1\right)$$

In the same way, the partial derivatives of each term of the state transition matrix are calculated and set to 0, we can get that:

(11) $$a_{ij}=\frac{\sum_{t=1}^{T-1}\xi_{t}\left(i,j\right)}{\sum_{t=1}^{T-1}\gamma_{t}\left(i\right)}\;\;i,j=1,\cdots ,N$$

The third term of Equation (2) has different forms of solution according to the distribution of output sequence. When the output sequence obeys the discrete distribution, the Lagrange function is:

(12) $$L_{3}\left(B,\theta^{old},\lambda_{1},\cdots ,\lambda_{N}\right)=\sum_{t=1}^{T}\sum_{i=1}^{N}p\left(q_{t}=s_{i},O|\theta^{old}\right)\ln b_{j}\left(o_{t}\right)+\sum_{j=1}^{N}\lambda_{j}\left(\sum_{k=1}^{M}b_{j}\left(k\right)-1\right)$$

The partial derivative of the function to *B* is juxtaposed to 0, we can get that:

(13) $$b_{j}\left(k\right)=\frac{\sum_{t=1,o_{r}=v_{k}}^{T}\gamma_{t}\left(j\right)}{\sum_{i=1}^{T}\gamma_{t}\left(j\right)}$$

If the output observations corresponding to HMM states obey Gaussian distribution $B=\left\{\mu_{i},\sum i\right\},i=1,\cdots ,N$, assume that each state corresponds to a Gaussian distribution, and *n* is the dimension of the output observations, then the number of output distributions and states is the same as *N*. The Lagrange function is written as:

(14) $$L_{3}\left(B,\theta^{old}\right)=-\frac{1}{2}\sum_{t=1}^{T}\sum_{i=1}^{N}p\left(q_{t}=s_{i},O|\theta^{old}\right)\left[n\;\;\ln\left(2\pi \right)+\ln\left|\Sigma_{i}\right|+\left(o_{t}-\mu_{i}\right)\right]$$

We can get the partial derivative of function to $\mu_{i}$ and ∑i and make the partial derivative 0:

(15) $$\mu_{i}=\frac{\sum_{t=1}^{T}\gamma_{i}\left(t\right)o_{t}}{\sum_{n=1}^{T}\gamma_{i}\left(t\right)}\;\;i=1,\cdots ,N$$

(16) $$\sum i=\frac{\sum_{t=1}^{T}\gamma_{i}\left(t\right)\left(o_{t}-\mu_{i}\right){\left(o_{t}-\mu_{i}\right)}^{T}}{\sum_{n=1}^{N}\gamma_{i}\left(t\right)}\;\;i=1,\cdots ,N$$

If the output sequence corresponding to the state of HMM model is represented by Gaussian mixture model distribution, $B=\left\{B_{km},\mu_{km},\sum km\right\}$, *N* are the number of States, and *M* is the number of Gaussian distributions contained in the Gaussian mixture model corresponding to the output sequence in each state. Then the probability of output corresponding to state *j* can be expressed as:

(17) $$B=\left\{B_{km},\mu_{km},\Sigma_{km}\right\}$$

Here we introduce the intermediate variable *v* and satisfy the following conditions:

(18) $$p\left(v_{m}|q_{i}\right)=B_{im},1⩽i⩽N;1⩽m⩽M$$

(19) $$v_{j}\left(o_{t}\right)=N\left(o_{t}|\mu_{jm},\Sigma_{jm}\right)$$

The constraint condition is as follows:

(20) $$\sum_{m=1}^{M}B_{jm}=1,\cdots ,N$$

Finally, the likelihood function can be written as:

(21) $$\begin{array}{cc} L_{3}\left(B,\theta^{old},\lambda_{1},\cdots ,\lambda_{N}\right)\; & =\sum_{t=1}^{T}\sum_{j=1}^{N}\sum_{m=1}^{M}p\left(v_{t}=v_{m},q_{t}=s_{j},O|\theta^{old}\right)\\ \; & \left[\ln B_{jm}-\frac{n}{2}\ln\left(2\pi \right)-\frac{1}{2}\ln\left|\Sigma_{jm}\right|-\frac{1}{2}{\left(o_{t}-\mu_{jm}\right)}^{T}\sum_{jm}^{-1}\left(o_{t}-\mu_{jm}\right)\right]\\ \; & +\sum_{j=1}^{N}\lambda_{j}\left(\sum_{m=1}^{M}B_{jm}-1\right) \end{array}$$

Because of the introduction of intermediate variables, the forward and backward algorithms including implicit variables are redefined:

(22) $$\alpha_{t}\left(im\right)=p\left(\nu_{t}=v_{m},q_{t}=s_{i},o_{1},\cdots ,o_{t}|B\right)$$

(23) $$\beta_{t}\left(im\right)=\sum_{j=1}^{N}\sum_{l=1}^{M}a_{ij}B_{jl}N\left(o_{t+1}|\mu_{jl},\Sigma_{jl}\right)\beta_{t+1}\left(jl\right)$$

(24) $$\eta_{t}\left(im\right)=p\left(v_{t}=\upsilon_{m},q_{t}=s_{i}|O,\theta \right)$$

Similarly, let the likelihood function calculate the partial derivative for each member of $B=\left\{B_{km},\mu_{km},\Sigma_{km}\right\}$ and set it to 0 to obtain the extreme value, and finally get that:

(25) $$B_{jm}=\frac{\sum_{t=1}^{T}\eta_{t}\left(jm\right)}{\sum_{t=1}^{T}\sum_{m=1}^{M}\eta_{t}\left(jm\right)}\;\;j=1,\cdots ,N;m=1,\cdots ,M$$

(26) $$\mu_{jm}=\frac{\sum_{t=1}^{T}\eta_{t}\left(jm\right)o_{t}}{\sum_{t=1}^{T}\eta_{t}\left(jm\right)}\;\;j=1,\cdots ,N;m=1,\cdots ,M$$

(27) $$\Sigma_{jm}=\frac{\sum_{t=1}^{T}\eta_{t}\left(jm\right)\left(o_{t}-\mu_{jm}\right){\left(o_{t}-\mu_{jm}\right)}^{T}}{\sum_{t=1}^{T}\eta_{t}\left(jm\right)}\;\;j=1,\cdots ,N;m=1,\cdots ,M$$

In this paper, the gait phase is divided into four phases based on the angular velocity collected at the ankle. In the process of the four stages transition, the states are from left to right in turn, and there is no jump transition. In this paper, the four gait stages are represented by the four states of Markov chain in HMM. The corresponding states of the four stages of gait can not be directly observed, but the angular velocity measured by the sensor. So in this paper, Gaussian distribution or Gaussian mixture distribution is used to realize the mapping relationship between the state and the output, and realize the division of gait stages.

## 4. Materials and Methods

Based on the data acquisition system, this section gives the overall scheme of gait phase recognition based on inertial sensors. The sensor data acquisition, feature extraction and gait phase division of the system are described.

### 4.1. Gait Data Collection

**Figure 3:**
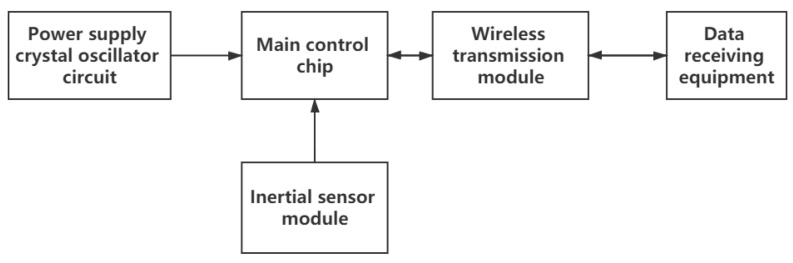
Data Acquisition System.

As shown in Figure 3, the data acquisition part consists of a minimum system composed of a main control chip and peripheral circuits, an inertial sensor module for gait signal sensing, a wireless signal transmission module and a data receiving device.

#### 4.1.1. Data Acquisition Hardware Platform

**Figure 4:**
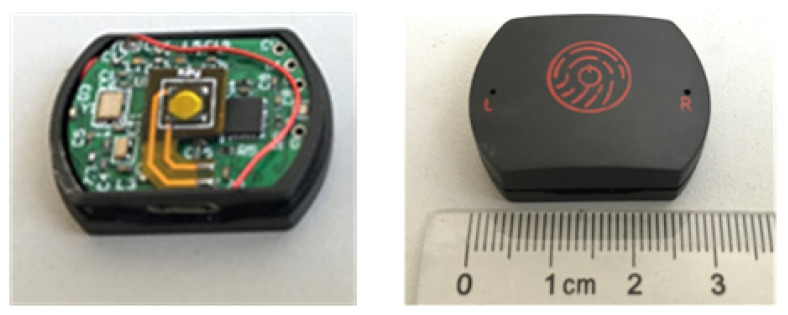
System hardware.

The selection criteria of data acquisition system usually include sensor accuracy, sensor drift and sampling frequency. With regards to the transmission module, it is always necessary to consider the transmission speed and transmission reliability as well as the convenience of practical use, and the gait behavior recognition acquisition system needs to meet the characteristics of small size and low power consumption of wearable devices as far as possible. Compared with image acquisition, the acquisition of angular velocity by inertial sensors is convenient and easy to operate, and due to the development of MEMS technology, the use of inertial sensors now has a low power consumption, small size, and lightweight and other superior performance. The sensor module is manufactured by Invensense (Sunnyvale, CA, USA). The accelerometer sensitivity is ±4800 LSB/g, and the range of accelerometer can be set as ±2, ±4, ±8, ±16 g. According to the needs of this research, the measuring range of the gyroscope range is set at $\pm 2000^{\circ }$ and the sampling frequency is 100 Hz. The developed system hardware is shown in Figure 4.

**Figure 5:**
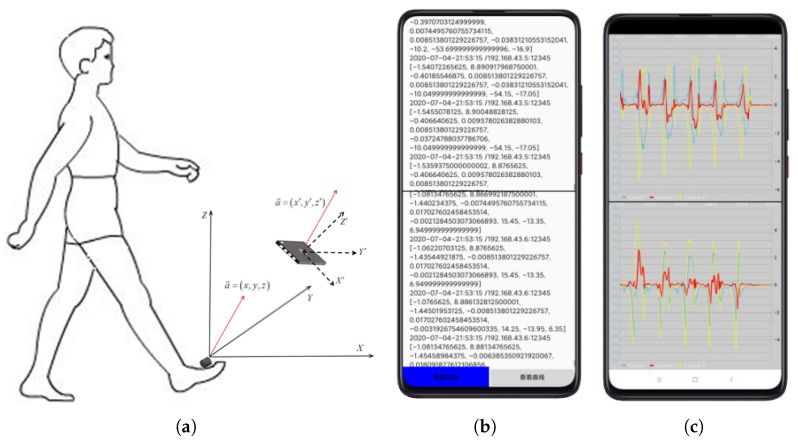
Data acquisition and software interface (**a**) Sensor placement (**b**) Raw data (**c**) Raw data curve.

Human gait signal collected by the system can be stored directly by adding memory in the system, or by wired and wireless communication mode. Each of these methods has its advantages and disadvantages. The advantages of wired mode are fast storage speed and low packet loss rate, the disadvantage is that the process of data storage is not observable and may be lost. Wired transmission is rarely used in practice, although it can have both fast transmission and real-time data visualization. The wireless transmission uses electromagnetic waves to send and receive signals for communication. For example, researchers usually use data collected simultaneously from the waist, thighs, calves, instep, toes and ankles or multiple parts to identify the gait stage. In this paper, a bandage is used to attach a wearable sensor to the human toe (as shown in Figure 5a) to capture the angular velocity generated by the human gait behavior, which is passed through Wi-Fi to the mobile phone.

#### 4.1.2. Software Platform

The data collected by the hardware circuit will be sent to the mobile phone through wireless transmission. Therefore, Wi-Fi wireless communication software based on the Android operating system is compiled. As shown in Figure 5b, the software can receive the data collected by the Wi-Fi signal transmitter in real-time during normal operation, and save the received data to a file. The data can be read to the computer through a USB serial port to facilitate the subsequent algorithm research, and the received signal change curve is displayed on the mobile phone. The collected original 3D angular velocity signal is shown in Figure 5c.

### 4.2. Gait Data Preprocessing

Smoothing and de-noising the collected raw data is an indispensable step in the data processing process. Human gait behavior is low-frequency motion, in this case, it is necessary to filter the mixed high-frequency noise. The ways of generating these noises are noise caused by relative movement between acquisition equipment and the human body; there will also be noise interference in the process of digital signal conversion; electromagnetic interference introduced in the process of data transmission from acquisition equipment to receiving equipment; noise generated by power supply circuit of acquisition equipment [42,43,44]. Most of the noise can be eliminated by preprocessing original data. The common data preprocessing methods include signal denoising, smoothing, and normalization. Data preprocessing can be done by using a filter circuit in the data acquisition system or by the software program. Butterworth filter, FIR low-pass filter, moving average filter, median filter, Wiener filter, and wavelet filter software filtering methods are commonly used.

**Figure 6:**
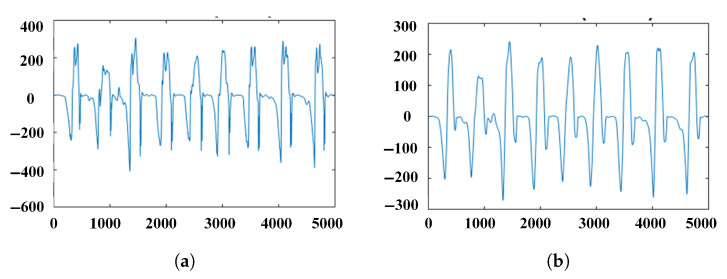
Angular velocity signal (**a**) Before preprocessing (**b**) After preprocessing.

The common data smoothing methods include moving average filtering, median filtering, three-point linear smoothing filtering, and five point linear smoothing filtering. The selection of filtering methods is often related to the similarity of the signals before and after filtering. It is more likely to judge which filtering method to use by observing the dynamic perception and identification of subjective factors of human motion imbalance state. The filtering method selected in this way can have a better effect, but it may not be the best method. Based on information theory, this paper proposes a selective filtering method by analyzing information contained in the signal. By comparing the signal-to-noise ratio (SNR) and root mean square error (RMSE), the optimal filtering method is selected by comparing the moving average filtering, median filtering, and five point cubic filtering. The effect of sliding filtering is related to the size of the sliding window, too large windows will lead to serious signal distortion and signal delay. By comparing the window size to 15, as shown in Figure 6a,b, the changing trend of angular velocity signal after filtering is more smooth, which can reduce most of the interference.

### 4.3. Window Segmentation for Gait Data

Window segmentation is the process of cutting a set of gait data according to the actual requirements and then extracting features from the data within the window as a recognition algorithm. The window is used as the basic unit of data in the feature extraction and gait phase identification process. There are three popular window classification methods: behavior-based window, event-based window, and sliding-based window [45,46,47,48,49]. The behavior-based window determines the location of the window segmentation in the raw data based mainly on the behavior change in the data; the event-based window determines the location of the window segmentation in the raw data through the specific events in the data; the sliding-based window segmentation method is similar to the previous two methods of determining the location of window segmentation, however, unlike the original data, the sliding-based window is not directly related to the raw data and it uses an equidistant window to partition the data.

**Figure 7:**
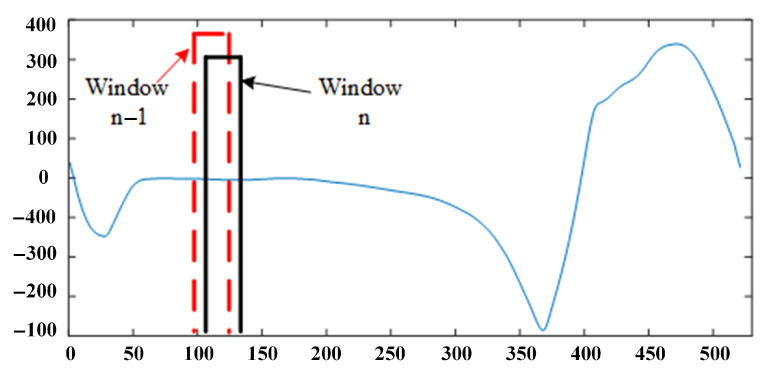
Using sliding window to split gait data.

Among the three window segmentation methods, the sliding-based window is well adapted to the periodic, stable, and some sporadically distributed behaviors [50]. Because the threshold of behavior-based and event-based segmentation needs to be set and adjusted according to different curves. As well as the interference signal will affect the threshold judgment and the gait changes periodically, the use of behavior-based and event-based window segmentation methods is not as effective as the sliding-based window segmentation method. In this paper, the sliding-based window segmentation method is used for both feature extraction and gait event recognition, and we test the number of overlaps of adjacent window. The size of the window needs to be determined based on factors such as the actual data type and sampling frequency, the larger the window size, the more pronounced the feature differences are, and the more latency is exhibited. The window size defined in this paper does not exceed the state that occupies the smallest percentage of the gait phase, to avoid a window of data containing multiple gaits states which leads to reduced stage recognition. A window which is too small extracts features that are not representative of the gait stage. The choice of the gait stage window size is a process of balancing recognition speed and recognition accuracy. The data segmentation method based on the sliding window is shown in Figure 7.

As mentioned previously, the principles and feasibility of the hidden Markov model for gait recognition were introduced, and the model was analyzed in the context of gait stage recognition. Different gait stage divisions and different sources of gait data make the structure of the Hidden Markov model different. A complete gait cycle can be divided into multiple gait stages according to practical requirements, and the states corresponding to the gait stages have Markov property, so the hidden Markov model is widely used in the field of gait stage classification and gait recognition.

Since the actual situation is far more complex than the ideal situation, there are many problems when using the hidden Markov model to solve the gait stage recognition problem, the main problem is that the three parameters of the hidden Markov model are fixed, and there is no way to adaptively handle the specific use case.

The gait motion signal is quasi-periodic, and although the motion gait is periodic, the length of the gait period, the length of a complete cycle factor such as the percentage of each stage, and the variation in data magnitude can be greatly disturbed in actual scene. At the same time, gait behavior is arbitrary and variable due to individual and environmental diversity, making gait signals (through various gait information acquisition devices to obtain kinematic, kinetic, and physiological information about gait) exhibit periodicity as well as uncertainty and nonlinearities, and complex and non-unique correlations, which add to the importance of period determination, phase delineation, similarities in gait stage analysis and identification.

Usually, the process of gait stage segmentation by hidden Markov models is to firstly estimate the parameters using certain data and then the parameters are brought into the model and the state sequence is then computed using the Vibbit algorithm, i.e., the trained model is used to identify the gait data of others. Good results can be obtained when gait data used for recognition and training are not very different. If the difference between the recognition data and the training data is large, the actual recognition accuracy will become very low. A simpler approach is to use a larger training data set to allow the model to cover a wider gait space. Although it may reduce the effect of the difference between the data used for training and the data used for recognition, it makes the gait phase recognition of the parameters widely distributed, which makes the performance of model recognition degrade. Another approach is to calculate a specific model based on a specific situation and select a specific model based on the actual situation during the use of the identification. This approach is also problematic in that gait staging based on a particular situation requires extensive analysis of movement data to do so; there is no explicit method to determine the gait for different situations, which in turn poses a more complex problem for gait stage identification.

It would be an interesting direction of research to start with the problem of gait modeling itself to determine a universally applicable method. Due to the quasi-periodic nature of human gait and the variability of human gait in different situations, a better approach is to use adaptive techniques. Adaptive approaches have been widely studied and used in solving speech recognition problems using hidden Markov models, and this research expects to put this technology applied in gait stage recognition, motion gait adaptation required correcting the model parameters with some of the gait data used for recognition. This makes the model parameters more suitable for gait recognition, to improve the accuracy and recognition rate of the hidden Markov model. The analysis from the model perspective enables the adaptive motion gait technology to realize the self-adaptive motion behavior of different individual in different environments with different movements.

Adaptive methods for models can be generally divided into two categories: feature layer-based adaptive methods and model layer-based adaptive methods. Adaptive methods based on the feature layer from the acquisition system to get the gait signal in different cases independent of the differences between the different cases of the feature extraction ensemble, i.e., the extracted features are not correlated with the factors causing the differences. The model layer-based adaptive method modifies the model parameters based on the differences between the actual measurements and the trained model so that they can be better used in the current identification and classification of actual data.

**Figure 8:**
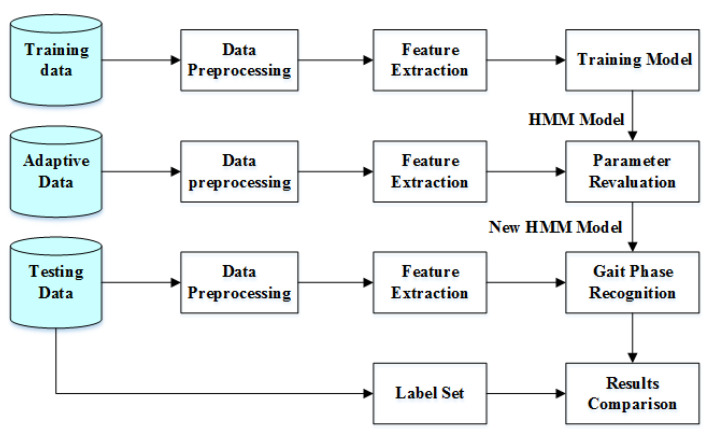
Flow chart of adaptive algorithm based on model layer.

As shown in Figure 8, the model layer-based adaptive approach is based on modifying the model parameters according to the actual gait signal to be identified. The gait stage recognition hidden Markov model parameters is calculated from the training data. After the parameter training is completed, if the parameters remain unchanged, since the model parameters are collected by a specific individual under specific conditions, so that during the recognition phase the results may vary considerably for different individuals under different conditions. To counteract the effect of the differences, one way is to use a large number of gait data collected from different individuals under different conditions at the same time during the training phase. The data is used to target train multiple gait stage recognition models, and the recognition stage selects the appropriate model from these models, or firstly using a small amount of data to substitute all models to select models with high recognition rates for gait stage recognition; the other way is to take these model parameters to generate new model parameters by linear combination, an approach that simply sums up the set of parameters in various cases, and failure to consider special cases can make the model parameters become too widely distributed rather than sharply distributed, so the recognition effect is neither too high nor too low.

The model-layer based adaptive approach is based on modifying the model parameters according to the actual gait signal to be identified. The model layer-based adaptation can be broadly divided into two categories depending on the algorithm: direct model adaptation algorithms (Direct Model Adaptation) and transform-based model adaptation methods (Transform-based Model Adaptation). Typical direct model adaptation algorithms have a Maximum a Posteriori (MAP), Minimum Classification Error (MCE), and Structural Maximum a Posteriori (SMAP), et al.

The model adaptive algorithms that directly adjust parameters can adjust those parameters for which there is a distribution of observed output in the adaptive data. More data is often required to achieve better adaptive results. Transform-based adaptive algorithms are maximum likelihood linear regression, maximum a posteriori linear regression, minimum classification error linear regression, etc. The conversion-based model adaptive algorithm obtains a series of linear transformations based on the differences between the source model and the target model to achieve a good adaptive effect on the model and can adjust all distribution parameters in the model. The adaptive algorithm can get better results with a small amount of data compared to the direct adjustment of the model parameters.

In this paper, MAP and MLLR are selected to adjust the parameters of the hidden Markov model for the human gait stage division. Bayesian theory, which combines a priori information about the data being adapted and the model parameters so that the model’s posterior probability is maximized. MLLR with transformation mechanism is used to transform the parameters of the model into a feature space that is close to the adapted data.

## 5. Experimental Results

In the previous sections, we have analyzed the algorithm principle and feasibility of the hidden Markov model in gait stage recognition and the process of solving the model parameters, as well as two common methods of adjusting the model parameters, which will be tested and verified in this section.

### 5.1. Experimental Data Source

#### 5.1.1. Data Collection Object

It is common for researchers to use, for example, the waist, thighs, calves, backs of feet, toes, ankles, or multiple sites to collect data simultaneously to perform gait phase recognition [16,51,52,53,54,55,56,57,58]. In this paper, a bandage is used to attach a wearable sensor to the human toe to capture the angular velocity generated by the human gait behavior, which is passed through Wi-fi to the mobile phone.

There were sixteen volunteers involved in the experimental data collection, eleven males and five females, ranging in age from 30 to 60 years old, height from 1.59 to 1.84 m, and weight from 49 to 88 kg. The data collection sensors were fixed on the both toes, and the angular velocity of gait during walking of normal volunteers was collected during the experiment. These volunteers were quite different in age and fluctuated in weight, which can be used to compare model accuracy before adaptation and after parameter adaptation.

#### 5.1.2. Gait Data Collection

During the collection of data from the inertial sensors that embody gait behavior, the angular velocity signals of eight volunteers during normal walking were collected, and each of the volunteers walked on level ground with their customary walking habits, and inertial data were collected twice for each volunteer, with the distance walked each time 11 m, removing the first and the last cycle that differs significantly from normal gait data during model training and gait phase identification gait signal. The sampling frequency of the data acquisition device is 100 Hz, the normal human walking speed is around 1 m/s, and the distance between each step is about 60∼75 cm, so the gait frequency is approximately one and a half steps per second, so the set sampling frequency can capture a complete sample of each gait phase.

### 5.2. Model Performance Evaluation Metrics

When evaluating the effectiveness of an analytical model for gait recognition, comparing the accuracy alone is not sufficient to fully evaluate the performance of the model. The problem of gait phase recognition in this paper can be seen as a binary classification problem, including both correct and incorrect recognition. Usually, dichotomous problems are classified by Precision (P), Recall (R), Sensitivity (True Positive Rate, TPR), Specificity (False Positive Rate, FPR), and F-Measure, which are evaluated by the confusion matrix. The horizontal coordinate of the P-R curve is the recall rate, and the vertical coordinate is the precision rate, and the ROC(Receiver Operator Characteristic) curve with specificity as the horizontal coordinate and sensitivity as the vertical coordinate. When the numbers of positive and negative samples in the data tested are similar, both ROC and P-R have good performances. However, if the negative sample number is larger, the ROC curve effect can still maintain the trend, while the effect on the PR curve is poor. The uesd Performance evaluation indicators are shown in Table 1.

The classes used for identification are usually called positive classes, denoted by 1, while the others are negative classes, denoted by 0. Four scenarios will emerge from the classification model’s identification of test data.

TP: The model identifies a positive sample as a positive sample.
FN: The model identifies a positive sample as a negative sample.
FP: The model identifies a negative sample as a positive sample.
TN: The model identifies a negative sample as a negative sample.

### 5.3. Analysis of Results Based on Hidden Markov Models and Improved Models

In previous sections, we have analyzed the algorithm principle and feasibility of the hidden Markov model in gait stage recognition and the process of solving the model parameters, as well as two common methods of adjusting the model parameters, which will be tested and verified in this section.

Since the information collected by the acquisition device is continuously changing, so the output signal of the hidden Markov model for human gait stage recognition is continuous, and the output corresponding distribution needs to be described by a continuous function, so this paper will verify the model performance when the corresponding output signal of the gait stage obeys two distributions respectively, i.e., the Gaussian and Gaussian mixed distribution.

#### 5.3.1. Recognition Results of HMM with Gaussian Distribution

**Figure 9:**
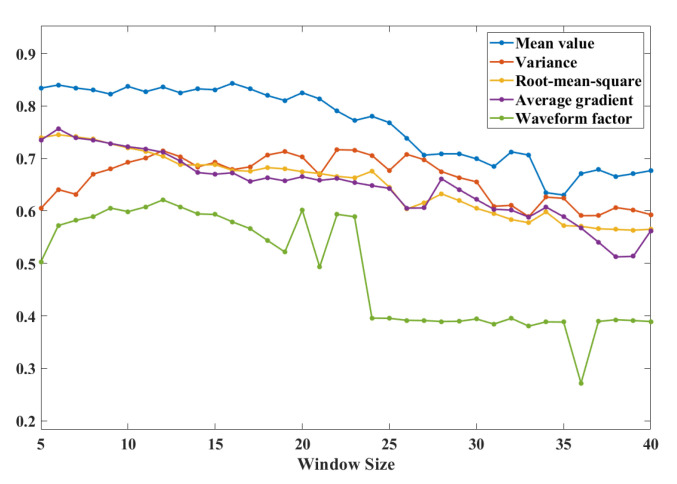
Change of eigenvalue recognition rate with sliding window size.

The appropriate size of the sliding window for each feature was first tested separately, and Figure 9 gives the values of some features. The recognition rate of the sliding window varies with the sliding window size. The size of the overlapping portion between adjacent sliding windows is taken to be half the size of the sliding window. The curve in Figure 9 lists the five eigenvalues: mean, variance, root mean square, mean gradient, waveform factor recognition rates and window size relationship, it can be seen that each feature takes the optimal value in the sliding window size range of 5 to 15 when combining multiple eigenvalues for gait stage recognition. One may use this range as a reference to find the most appropriate window within the range size which can shorten the time it takes to find a model.

**Figure 10:**
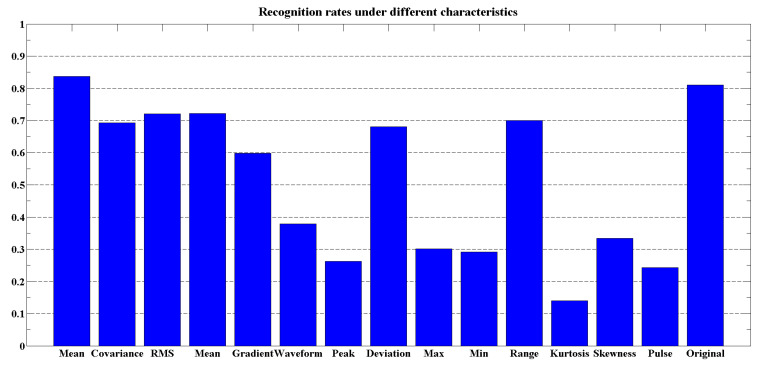
Recognition rate of eigenvalues with sliding window size of 10.

In Figure 10, the recognition rates of 15 common time-domain features are shown with the sliding window size set to 10, from which all the mean value of raw value, root mean square (RMS), and absolute mean reach the recognition rate of 70% and above. When using multidimensional features for gait stage recognition, these features should be given priority.

**Figure 11:**
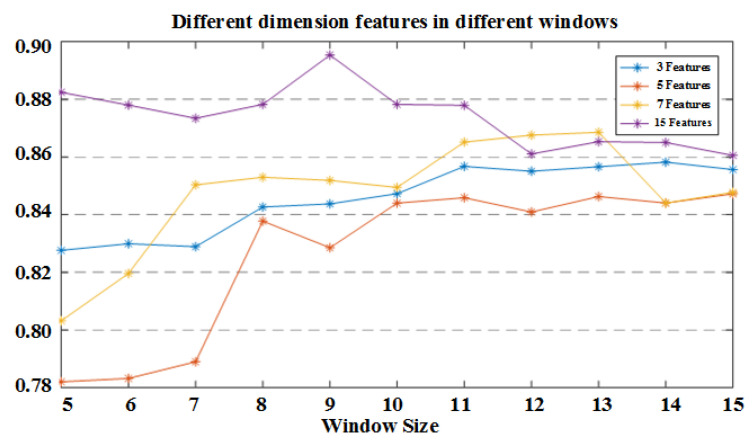
The recognition rate of different feature dimensions under different sliding windows.

Previously, features of individual dimensions were analyzed for gait stage recognition rates for different size windows and different features, and Figure 11 draws the trend of recognition rate with the sliding window size after combining multiple features into a multidimensional feature. The recognition rate of the eigenvalues in Figure 9 is selected from the largest to the smallest. For example, the 3D features selected in the figure are the mean, the raw data sampling values, the average of the absolute values, the five-dimensional and seven-dimensional features are also selected according to this rule. The optimal sliding window size for gait stage recognition using 3D features is 14 and the recognition rate is 85.82%; four-dimensional features had an optimal recognition rate of 84.59% at a sliding window size of 12. Seven-dimensional features have the best recognition rate of 86.86% at a window size of 14; When the feature size is increased to 15, the best sliding window is 9, corresponding to the gait phase recognition rate of 89.54%.

#### 5.3.2. Recognition Results of HMM with Gaussian Mixed Distribution

The Gaussian mixed distribution is more likely to contain the distribution of the output of the corresponding state of the gait phase than the distribution whose output is Gaussian. When the hybrid Gaussian model is used as the output distribution of the hidden Markov model identified by the gait stage, assuming that each state corresponds to the mixed Gaussian model and contains the same number of Gaussian distributions, assuming it was found previously that when the feature dimension is 15, the highest gait stage recognition rate can be obtained, and therefore no longer uses unidimensional features as observations for the gait stage recognition model in this section.

**Figure 12:**
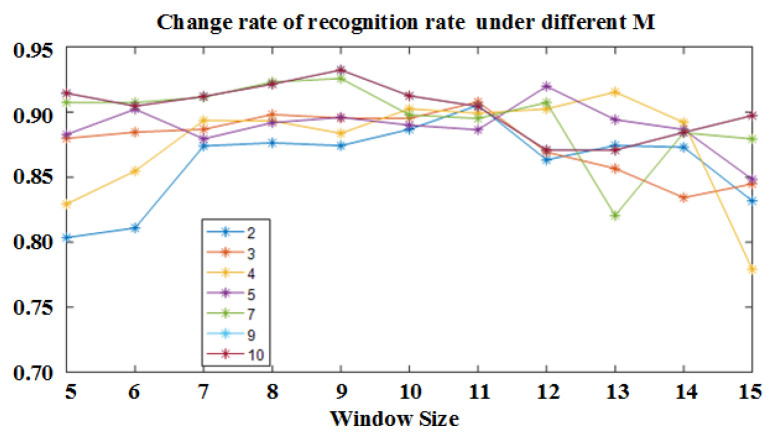
Change rate of recognition rate with sliding window under different M.

Figure 12 shows the recognition rate of the gait stage recognition model with a sliding window size in the range of 5 to 15 when M is taken at different values. Each curve in the figure corresponds to the recognition rate trend, which firstly goes up and then goes down, the appropriate window range is roughly between 9 and 15. It can be seen from the figure that the trend of the recognition rate increases gradually as the value of M increases to the highest recognition rate. Considering the influence of model parameter complexity on the time complexity of the subsequent state sequence calculations, the Gaussian hybrid model is used when the output distribution of the stage is distributed, we selected the recognition effect of the model when M is 5 and the sliding window size is 12 for comparison.

### 5.4. Identification Results after Model Parameter Adaptation

#### 5.4.1. Performance Analysis of Gaussian HMM after Parameter Adaptation

It can be seen from Figure 10 that the highest recognition rate is onbtained when the unidimensional feature is the mean. Taking the mean as an example, it is meaningful to analyze maximum likelihood linearity regression estimation and maximum a posteriori probability estimation of the effect of parameter adaptation of the model on recognition rates. Table 2 presents the parameters before and after model adaptation. Since this paper assumes sequential transitions between gait stages, the initial state probabilities will no longer be included in the table, and the parameters used in this paper are obtained from the adaptation algorithm by adjusting the mean and variance, and the table shows the mean and variance of the parameters before and after adaptation.

The state transfer matrix A is a 4 by 4 square with the same state transfer matrix for all three models as shown in the following equation.

$$A=\left[\begin{array}{cccc} 0.8460 & 0.1540 & 0 & 0\\ 0 & 0.8253 & 0.1747 & 0\\ 0 & 0 & 0.7395 & \;0.2605\\ 0.1649\; & 0 & 0 & 0.8351 \end{array}\right]$$

The experiments in this paper all based on the assumption that each experiment starts from a state where the heel is off the ground, so the initial probability matrix is shown as:

$$\pi =\left[\begin{array}{cccc} 1 & 0 & 0 & 0 \end{array}\right]$$

**Figure 13:**
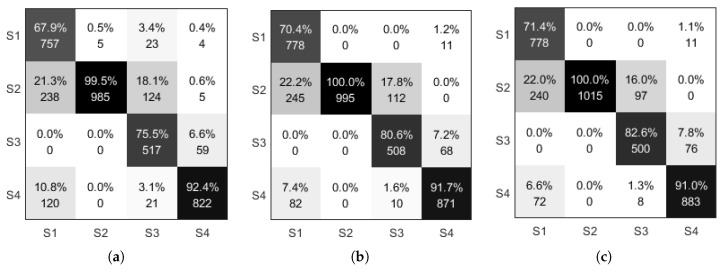
Confusion matrix for model recognition (**a**) HMM (**b**) MLLR (**c**) MAP.

From Figure 13, it can be seen that the number of correctly recognized state sequences for states S1, S2, and S4 has increased considerably. There is a smaller decrease in the number of correct identifications for state S3, but the overall identification rate has increased.

#### 5.4.2. Performance Analysis of Gaussian Mixed HMM with Adaptive Parameters

After obtaining the effect of the number of Gaussian distributions M and the size of the sliding window on the recognition rate in the Gaussian mixed model. The Gaussian distribution can be viewed as a special case of mixed Gaussian distributions, the discussion of parameter adaptation model in this section is based on the output of the gait stage recognition model which obeys the basis of the hybrid Gaussian model.

**Figure 14:**
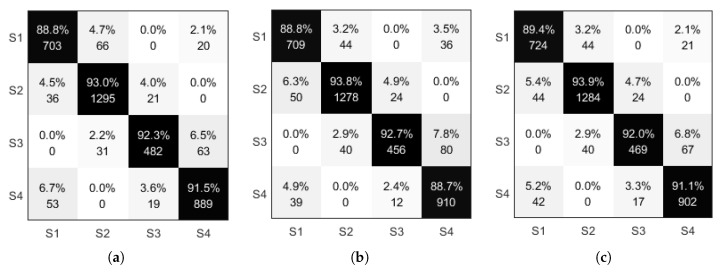
Confusion matrix of the model before and after parameter adaptation (**a**) HMM (**b**) MLLR (**c**) MAP.

This section presents the MAP and MLLR based algorithm. The results after parameter adaptation are compared with the identification model of the gait stage with unadjusted parameters merely using the correct identification state. The ratio of the number of states to the total number of states used for identification is not comprehensive enough to evaluate the model, so this section evaluates the gait stage model by using the confusion matrix. The model before and after parameter adaptation is compared, as shown in Figure 14, with the horizontal axis in the confusion matrix indicating the original state of the data and the vertical coordinate indicates the identified state.

As shown in the data in Figure 14a,b, the number of correct identifications of states S1 and S4 by maximum likelihood linear regression is greater than the number of states corresponding to correct recognition when the model is not adapted, and the opposite is true for states S2 and S3, where the number of states corresponding to correct recognition is less than the number of states corresponding to correct recognition when the model is not adapted. The confusion matrix of Figure 14c results from parameter revaluation using the maximum posterior probability estimated a priori information about the model parameters, so this paper adjusts the maximum a posteriori estimation by the maximum likelihood linear regression method of the mean and variance obtained which are used as a priori information. The maximum a posteriori probability estimate combined with the model identification information obtained from the maximum likelihood linear regression can be seen summarized as follows: the number of correct identifications for S1 is higher than the first two methods; the number of correct identifications for states S2 to S4 is intermediate between the first two methods position, as the maximum, a posteriori estimate combines the original model with the parameters of the adapted maximum likelihood linear regression in the form of weights. It is theoretically normal that the iterative result does not exceed the highest of the two.

From the performance metrics in Table 3, it can be seen that when the output of the gait stage recognition model is Gaussian mixed distribution, relative to the single Gaussian distribution univariate and the recognition rate is improved when multidimensional features are present. When the output signal features of the model obey a Gaussian mixed distribution, maximum likelihood linearity when comparing the accuracy of the model before and after parameter adaptation and the parameter adjustment scheme combining regression and maximum a posteriori probability estimation results in an increase in the overall identification rate, and for the lower correct identification in the original model state, the recognition rate increases after parameter adjustment; meanwhile, the recognition rate is close to unadjusted after parameter adjustment with higher correct identification in the original model state.

**Figure 15:**
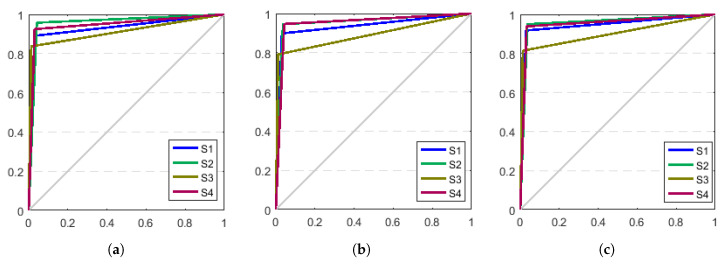
ROC curve of HMM parameters before and after adaptation (**a**) HMM (**b**) MLLR (**c**) MAP.

The ROC curves in Figure 15 for the three models show that the parameters of the maximum likelihood linear regression are used as the maximum posterior probability estimates to obtain the overall performance that is higher than when no parameter adaptation and maximum likelihood linear regression estimation are performed. As shown in the data in Table 3, the highest recognition accuracy of the gait stage model based on the angular velocity signal is around 92%, and the performance is not significantly higher after the adaptation of the model parameters.

The possible reasons for the lift of the gait phase are: first, overlapping data between adjacent windows at the time of feature extraction, close to the moment of gait phase transition stage judgments will be subject to errors; second, the hidden Markov model may be used for parameter adjustment schemes and there are differences in the speech recognition domain and gait stage recognition.

### 5.5. Comparison with Other Algorithms

**Figure 16:**
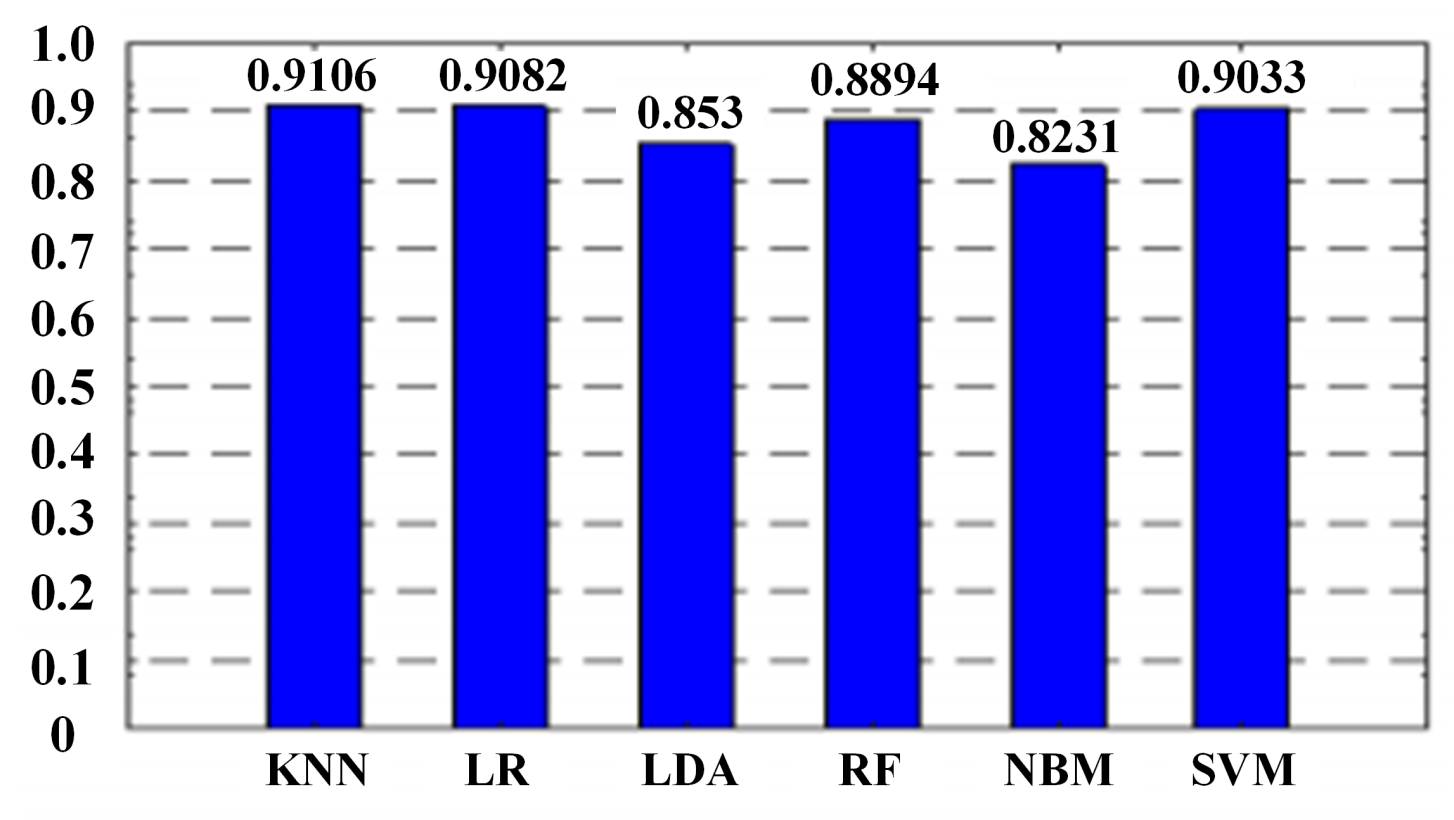
Comparison of recognition rates of different classification algorithms.

In order to test the advantages of HMM and the improved algorithm, this paper uses other six commonly used algorithms as a comparison: K-Nearest Neighbor(KNN), Logistic Regression(LR), Linear Discriminant Analysis(LDA), Random Forest(RF), Naive Bayesian Model(NBM) and SVM. The experimental results are shown in Figure 16. The resolution of the classification method is between 80% and 92%, which is close to the recognition rate of the HMM model. If only from the perspective of recognition rate, KNN, LR, and SVM can replace HMM as a model of gait cycle stage division. However, the gait phase division not only identifies specific stages but also needs to consider the transition relationship between states on time series. As a comparison, the six algorithms do not consider the transition between states, which may lead to state transition sequence errors, and then judge the number of cycles and the proportion of each state in each cycle proportion. The recognition rates of these six algorithms are shown in Figure 16.

**Figure 17:**
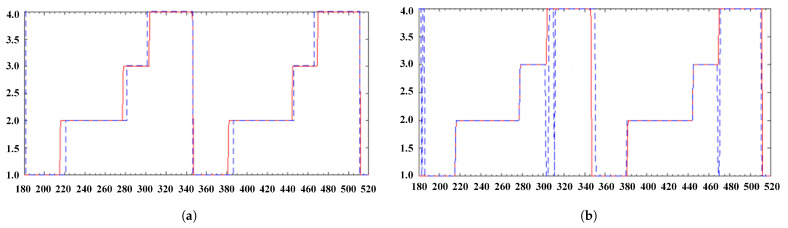
State sequence (**a**) Recognized by HMM (**b**) Recognition sequence of KNN.

Figure 17a,b show the state sequence recognition diagram of the improved HMM model and KNN respectively. The red solid line in the figures is the state sequence as the control, and the blue dotted line is the time series actually recognized by the model. It can be seen from the figures that the recognition error state mainly occurs near state transition time. In Figure 17a, the recognition error of the improved HMM model is to identify the state as the previous or the next state of the current state, which is determined by the state transition matrix. Therefore, there is almost no error in judging the number of cycles contained in a complete gait cycle. The length of the recognition error is about five time points, which is close to the coincidence length of two adjacent windows. The HMM result is acceptable because the minimum unit to distinguish the state of the HMM model is the length of a sliding window and adjacent windows. In addition, there will be errors in the state sequence compared with the state recognition, so the recognition error of the HMM model is inevitable. In Figure 17b, state S1 occurs in the process of state S3 and S4 transition, which has an impact on the number of cycles, cycle length, and the proportion of each stage. The same phenomenon also occurs in the other five algorithms during algorithm testing.

### 5.6. Analysis of Human Balance Ability by Gait Parameters

**Figure 18:**
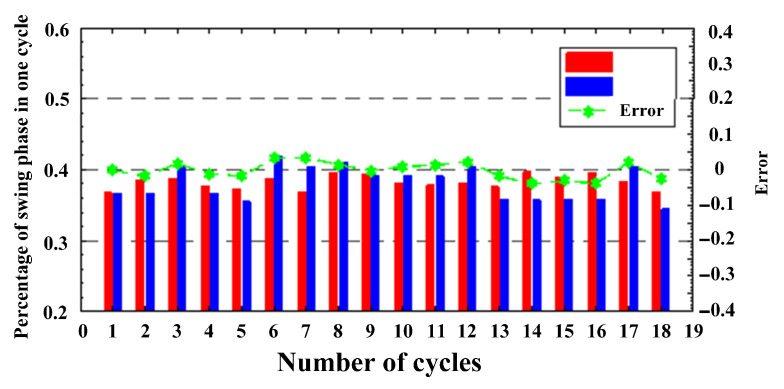
The ratio of the swing phase to the complete cycle.

The swing phase of normal people accounts for about 40% of the whole gait cycle. Patients with abnormal gait usually have shorter support phase and longer swing phase. The swing phase in the gait cycle corresponds to the state S2 of the HMM model in this paper. The red and blue histograms in Figure 18 show the proportion of the swing phase recognized by the control group and HMM models in the complete cycle, and the green curve represents the error between them. The maximum error accounts for 3.97% of the complete cycle and the average value is 2.02%, which proves the feasibility of HMM when dealing with gait cycle analysis.

Table 4 shows the spatiotemporal parameters related to human balance of the left and right lower limbs(LLL,RLL) of a normal person. The recognition results of HMM are very close to the control group results, and the left-right ratio of swing time, stance time to gait cycle reach 0.9453, 0.9241 and 0.9936 and 1.0000, respectively, indicating that the subject maintained good balance, which is similar to the control group results.

### 5.7. Experimental Conclusions

In this section, the actual effects of gait phase recognition models are compared. Firstly, the data source and data acquisition scheme are introduced; secondly, the indexes and curves for evaluating the algorithm are introduced; then, the overall recognition rate of single dimensional and multi-dimensional features before and after the model parameters are adapted and the recognition rate when the output is subject to different distribution are analyzed; compared with the recognition rate of the model before and after the parameter adjustment, the model parameters result in better perforamance. The recognition rate of the model is 91.88%, even though the age, height, and weight of the participants are different. At the end of this chapter, the gait parameters related to the balance ability of the human body are analyzed. The gait comparison parameters calculated according to the gait stages are consistent with the reference values. The limitations of the previous proposed Hidden Markov Model-based method mainly lie in that HMM depends only on each state and its corresponding observer. HMM models are memoryless and cannot take advantage of contextual information. Because it’s only related to the previous state, if we want to take advantage of more known information, we must build a high-level HMM model. We have been trying to address this recognized issue in this research, though much work remains.

## 6. Discussions and Conclusions

Gait analysis has been widely used in disease diagnosis, orthopedic surgery, and rehabilitation training in recent years, so it is more and more important to establish an accurate and effective walking model. One of the technical challenges in our previous work is the overflow problem of algorithm recursion, so we normalized the eigenvalues. Secondly, we have screened to extract the characteristic signal, and marked the phase division effect on some data. When the angular velocity signal has interference, the effect of maintaining regularity is better. In this paper, the data source and data acquisition scheme based on the inertial sensors are proposed, and the index of the evaluation algorithm is given. The segmentation of gait with the hidden Markov Model(HMM) is used to analyze the global recognition rate of single dimension and multi-dimension features before and after the model parameters are adaptive. Through analysis and comparison, the method has a high recognition rate, ensures the integrity and objectivity of gait data, and provides a new theoretical basis for medical diagnosis. This research tested different methods and the experimental results showed that MAP and MLLR achieved best performance with regard to parameter adaptation. In future work, we plan to conduct more comparative trials and try to find the deep mechanism of what kind of factors affect the performance of parameter adaptation. Deep learning models including CNN and RNN have been applied to clinical gait analysis applications, however, these methods require a lot of computation and the Real-time monitoring cannot be guaranteed. We will try to address this issue in future work.

## Figures and Tables

**Table 1:** Performance evaluation indicators.

Index	Formulation	Definition
Accuracy rate (*A*)	$A=\frac{TP+TN}{TP+FP+TN+FN}$	The percentage of samples correctly identified as a percentage of the samples used for testing indicates the overall identification rate of the system.
Recalling rate (*R*)	$R=\frac{TP}{TP+FN}$	The ratio of correctly identified positive samples in all positive, indicating the identification rate ofindividual states of the HMM model.
Precision ratio (*P*)	$P=\frac{TP}{TP+FP}$	The proportion of correctly identified positive samples in all positive samples, showing the effect of sample distribution on recognition rate.
F1 value	$F1=\frac{2TP}{2TP+FP+FN}$	The reconciled mean of *P* and *R* was combined to evaluate P and R.
Sensitivity (TPR)	TPR=R	Sensitivity is the same as recalling rate.
False accuracy (TPR)	$FPR=\frac{FP}{FP+TN}$	TPR is predicted to be the ratio of the positive sample to the negative sample.
ROC Curve	Not applicable	The larger the area enclosed by the curve, the higher the classification rate.

**Table 2:** Comparison of performance indicators.

Parameters	Mean Value	Variance
HMM	[−2.6518 3.7528 −1.0578 −0.1528 ]	[3.9164 2.4664 0.7880 0.0146]
MLLR	[−2.4168 3.6096 −1.3194 −0.2117]	[3.0880 2.2895 0.8294 0.0184]
MAP	[−2.3472 3.5249 −1.5249 −0.2398]	[2.7750 2.2851 0.7171 0.0192]

**Table 3:** Comparison of performance using different methods.

Performance Index	Without Parameter Adaptive	MLLR	MAP
**Accuracy rate (A)**	91.59%	91.16%	91.88%
**Precision ratio (P)**	0.8876, 0.9303, 0.9234, 0.9146	0.8885, 0.9383, 0.9268, 0.8869	0.8938, 0.9386, 0.9196, 0.9111
**Recalling rate (R)**	91.59%	91.16%	91.88%
**Precision ratio (P)**	0.8910, 0.9578, 0.8368, 0.9251	0.8986, 0.9453, 0.7917, 0.9469	0.9176, 0.9497, 0.8142, 0.9386
**F1 value**	0.8893, 0.9439, 0.8780, 0.9198	0.8935, 0.9418, 0.8539, 0.9160	0.9056, 0.9441, 0.8637, 0.9247
**Sensitivity (TPR)**	0.0308, 0.0424, 0.0127, 0.0307	0.0309, 0.0363, 0.0113, 0.0437	0.0300, 0.0364, 0.0129, 0.0327

**Table 4:** Comparison of gait spatiotemporal parameters.

	Reference Value of LLL	Reference Value of RLL	Recognition Value of LLL	Recognition Value of RLL
**Swing time (s)**	0.5879±0.0321	0.5954±0.0446	0.5864±0.0536	0.5543±0.0857
**Standing time (s)**	0.4093±0.0807	0.3977±0.0423	0.4079±0.0182	0.4414±0.0129
**Gait cycle (s)**	1.5636±0.1064	1.5746±0.3254	1.5607±0.1993	1.5707±0.3293

## Data Availability

Data available in a publicly accessible repository that does not issue DOIs. Publicly available datasets were analyzed in this study. This data can be found here: [http://lis.dlut.edu.cn/database_en.htm].

## Abbreviations

The following abbreviations are used in this manuscript:

MEMS	Micro-electro-mechanical Sensor
BSN	Body Sensor Network
IMU	Inertial Measurement Unit
ZUPT	Zero Velocity Updating
HMM	Hidden Markov Model
